# Volatile fatty acids as sustainable feedstocks for tailored microbial lipid production

**DOI:** 10.1186/s13068-026-02814-x

**Published:** 2026-08-28

**Authors:** Marieke Willing, Max Schneider, Mariia Kornilova, Michel Weber, Dania Awad, Felix Melcher, Michael Paper, Marion Ringel, Daniel Garbe

**Affiliations:** Werner Siemens-Chair of Synthetic Biotechnology, Technical University of Munich (TUM), TUM School of Natural Sciences, Lichtenbergstr. 4, 85748 Garching, Germany

**Keywords:** *Cutaneotrichosporon oleaginosus*, Oleaginous yeast, Single-cell oil, Volatile fatty acids, Odd-chain fatty acid, Fatty acid methyl ester, Fatty acid profile, Waste valorization, Circular bioeconomy

## Abstract

**Background:**

Interest in single-cell oils from oleaginous yeasts is growing with respect to their potential as sustainable alternatives to plant- and fossil-based oils. The yeast *Cutaneotrichosporon oleaginosus* is a promising candidate for microbial lipid production due to its broad substrate range and high lipid accumulation capacity. Among potential feedstocks, volatile fatty acids (VFAs) derived from organic waste streams have attracted increasing attention as low-cost, sustainable carbon sources. These VFA mixtures typically contain C2–C6 volatile fatty acids, including acetic, propionic, butyric, valeric, isovaleric, and caproic acids. However, a systematic understanding of how individual VFAs affect growth, lipid accumulation, and fatty acid composition in *C. oleaginosus* is still lacking.

**Results:**

This study investigates the effects of individual VFAs in fed-batch fermentations with *C. oleaginosus* ATCC 20509 under controlled, elemental carbon-equivalent conditions, using acetic, propionic, butyric, valeric, and caproic acid as well as the branched-chain acids 2-methylpropionic, 2-methylbutyric, and 3-methylbutyric acid. It was demonstrated that carbon chain length strongly influences these parameters, with growth and lipid production generally decreasing as chain length increases. The highest lipid titers were observed in acetic acid- and butyric acid-based fermentations. Even-chain VFAs primarily resulted in the synthesis of even-chain fatty acids. In contrast, odd-chain VFAs promoted the formation of odd-chain fatty acids, with propionic acid emerging as a particularly promising substrate for the targeted production of odd-chain fatty acids. Fermentations with branched VFAs revealed that growth and lipid accumulation are strongly reduced, potentially due to steric effects associated with methyl substituents. However, the presence of the methyl group did not significantly alter the overall fatty acid profile compared to unbranched carbon chains, as confirmed by NMR analysis.

**Conclusion:**

These findings demonstrate that the choice of substrate has a major impact on targeted lipid production. By enabling the modulation of lipid composition and supporting efficient biomass and lipid accumulation, volatile fatty acids offer a high-value alternative to conventional carbon sources. Overall, these results highlight the potential of VFAs to contribute to a circular bioeconomy by converting low-value waste streams into valuable microbial oils.

**Graphical Abstract:**

**Figure:**
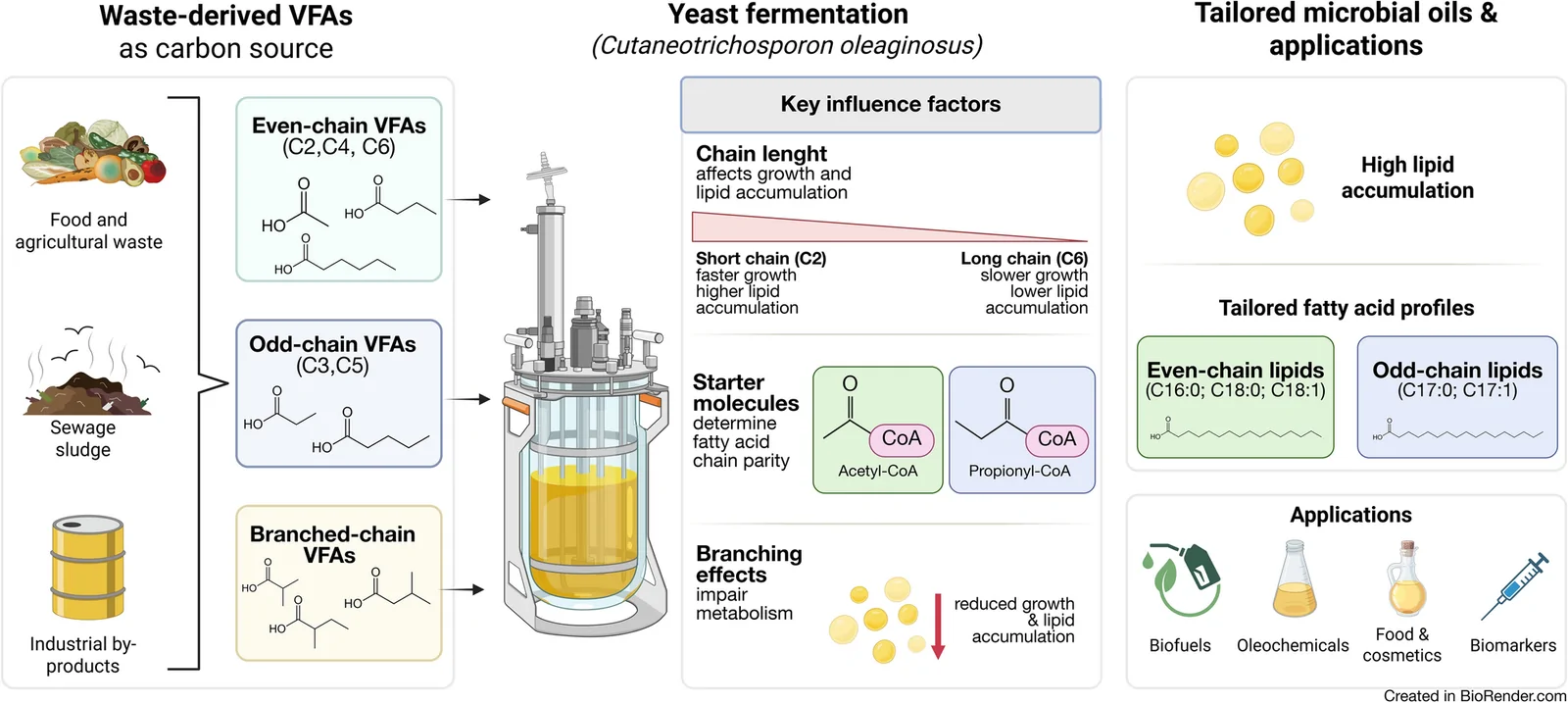

**Supplementary Information:**

The online version contains supplementary material available at https://doi.org/10.1186/s13068-026-02814-x.

## Background

In the context of a growing global population and the increasing demand for energy and food, interest in microbially produced oils, such as single-cell oils (SCOs), has risen considerably. Unlike fossil-based oils, which are inherently non-renewable and associated with significant environmental impacts, and plant-derived oils, which depend on land- and water-intensive cultivation and are influenced by geopolitical factors, climate conditions, and seasonal variability, SCOs offer a more flexible and scalable alternative [1, 2]. Microbial lipids can be utilized across a wide range of industries, including the food sector, biofuel production, cosmetics, and oleochemical applications [3–6].

For the production of SCOs, the oleaginous yeast *Cutaneotrichosporon oleaginosus* represents a particularly promising organism. This yeast can accumulate high levels of lipids, primarily triacylglycerides (TAGs), exceeding 60% (w/w) of its dry cell weight in the wild type [7, 8]. Moreover, *C. oleaginosus* exhibits remarkable metabolic flexibility and can utilize a broad spectrum of carbon sources, including sugars such as glucose, galactose, mannose, xylose, lactose, and *N*-acetylglucosamine, as well as alternative substrates like acetic acid and glycerol [9–13]. Among the alternative carbon sources investigated for *C. oleaginosus*, acetic acid is particularly well established as a non-sugar substrate. It can be efficiently applied in pH-controlled fed-batch fermentations and is readily converted to acetyl-CoA, the direct precursor of fatty acid biosynthesis, thereby enabling high lipid titers [11, 14]. However, beyond acetate, other volatile fatty acids (VFAs) offer additional potential to influence lipid metabolism. In particular, odd-chain VFAs such as propionic acid promote the formation of odd-chain fatty acids, demonstrating that substrate structure directly affects fatty acid composition and thus the functional properties of the resulting microbial oil [15, 16].

VFA mixtures are generated during the aerobic digestion of organic waste streams such as sewage sludge, food and agricultural waste, and typically consist of mixtures of acetic acid, propionic acid, butyric acid, valeric acid, isovaleric acid, and caproic acid [17–21]. The recovery potential of these VFAs is substantial. For example, dairy industry wastewater in the EU is estimated to yield up to 10 Mt/year, corresponding to approximately 2 Mt/year of acetate, 1.28 Mt/year of butyrate, and 1.54 Mt/year of propionate. On a global scale, the recovery potentials are estimated at 9.15 Mt/year for acetate, 5.39 Mt/year for butyrate, and 6.47 Mt/year for propionate, highlighting the significant potential of organic waste streams as sustainable carbon sources [22]. Such large quantities further demonstrate the feasibility of using VFAs as substrates for industrial-scale fermentation processes.

Despite these large quantities, VFA mixtures are currently used predominantly for methane production in biogas plants, a process that is comparatively inefficient in terms of carbon valorization [23]. Conventional biogas typically contains 50–75% CH₄ and 25–50% CO₂, with the CO₂ fraction commonly being released rather than further utilized. In addition, methane losses of 1–2.5% on average and up to 7.7% in wastewater treatment plants further decrease overall carbon recovery efficiency and represent a significant environmental concern due to methane's high global warming potential [24]. In contrast, the biotechnological conversion of VFAs into single-cell oils represents an efficient and high-value utilization pathway, enabling the production of tailored microbial lipids from low-value waste-derived carbon streams [25].

Previous studies have demonstrated the feasibility of using acetate, propionate, and butyrate as carbon sources for microbial lipid production [26, 27]. Moreover, investigations of VFA mixtures have shown that their composition strongly influences lipid yield and fatty acid profiles [28, 29]. However, these previous studies have largely focused either on VFA mixtures or on a limited number of individual VFAs, making it difficult to distinguish the specific contribution of each VFA to biomass formation, lipid accumulation, and fatty acid composition. In addition, most studies have been performed in shake flasks, whereas systematic comparisons of individual VFAs under controlled bioreactor conditions are still scarce. A systematic comparison of individual linear and branched VFAs under identical fermentation conditions is still lacking. To address this gap, the present study provides a comprehensive evaluation of acetic acid, propionic acid, butyric acid, valeric acid, caproic acid, isobutyric acid (2-methylpropionic acid), 2-methylbutyric acid, and isovaleric acid (3-methylbutyric acid) as carbon sources in pH–stat-controlled fed-batch bioreactor fermentations using a carbon-equivalent, consumption-based feeding strategy. This standardized cultivation approach [13, 14, 30] enables the direct comparison of individual VFAs under identical process conditions, thereby minimizing process-related variability. This experimental design enables direct assessment of the effects of carbon chain length, carbon chain parity, and methyl group position on biomass formation, lipid accumulation, and fatty acid composition. Through the systematic analysis of these structural factors, this study provides fundamental insights into the structure–function relationships of individual VFAs and their impact on microbial lipid production. Furthermore, the results highlight the potential of VFAs as versatile feedstocks for tailored fatty acid production and efficient microbial oil synthesis, thereby supporting the development of sustainable bioprocesses within a circular bioeconomy.

## Methods

### Cultivation

#### Strain and preculture

Cultivation was performed using the oleaginous yeast *Cutaneotrichosporon oleaginosus* ATCC 20509 (DSM-11815), obtained from the Deutsche Sammlung von Mikroorganismen und Zellkulturen (Braunschweig, Germany). A single colony of *C. oleaginosus* from a YPD agar plate was used to inoculate 100 mL of YPD medium (10 g/L yeast extract, 20 g/L peptone, 20 g/L glucose) in a 500-mL baffled Erlenmeyer flask containing antibiotics (0.1 g/L ampicillin, 0.05 g/L kanamycin). These precultures were incubated in a rotary shaker at 120 rpm and 28 °C for three days.

#### Medium composition

For fermentations using acetic acid as the carbon source, the medium composition was adjusted according to Rerop et al. [13] as follows: 30 g/L glucose, 4.5 g/L CH_3_COO∙Na, 3 g/L peptone, 3 g/L yeast extract, 3 g/L urea, 0.9 g/L Na_2_HPO_4_, 2.4 g/L KH_2_PO_4_, 2 g/L MgSO_4_·7H_2_O, 0.00055 mg/L ZnSO_4_·7H_2_O, 0.024 mg/L MnCl_2_·6H_2_O, 0.025 mg/L CuSO_4_·5H_2_O, 0.027 mg/L C_6_H_8_O_7_·Fe·H_3_N, and 0.1 mg/L thiamine. If a different acid was tested, the corresponding sodium salt replaced sodium acetate and was added in an amount providing the same total carbon content as sodium acetate. The required amounts of sodium salts were calculated based on their carbon content and resulted in the following concentrations: sodium acetate (4.5 g/L), sodium propionate (3.5 g/L), sodium butyrate (3.0 g/L), sodium valerate (2.7 g/L), sodium caproate (2.5 g/L), sodium 2-methylpropionate (3.0 g/L), sodium 2-methylbutyrate (2.7 g/L), and sodium 3-methylbutyrate (2.7 g/L).

#### Fermentation in parallel bioreactors

For the fermentations a DASbox 12-fold parallel bioreactor system (Eppendorf AG, Hamburg, Germany) with a maximum working volume of 250 mL and a total volume of 350 mL was used. The temperature was maintained at 30 °C, the pH at 5.7, and the dissolved oxygen (DO) at 10%, according to Schneider et al. [14]. These conditions were kept constant throughout the entire fermentation. The combination of 30 °C and pH 5.7 was selected based on Schneider et al. [14] demonstrating the highest lipid titers for *C. oleaginosus* under these cultivation conditions. Furthermore, *C. oleaginosus* is an acid-tolerant, obligately aerobic oleaginous yeast; therefore, maintaining a mildly acidic pH and sufficient oxygen availability supports efficient biomass formation and lipid accumulation. Initially, the pH was adjusted with 2 M HCl and 2 M NaOH to ensure the same starting conditions for all tested acids. No additional pH adjustment was performed after VFA addition, as pH was automatically controlled throughout the fermentation using the bioreactor control system. The initial stirring speed was set to 200 rpm and subsequently increased to 650 rpm as the first step of the dissolved oxygen (DO) cascade. In the next step, the oxygen fraction in the inlet gas was raised from 21 to 100%, followed by an increase in the aeration rate from 4.75 to 23 sL/h. To prevent foaming, Antifoam 204 (Merck, Darmstadt, Germany) was used. During fermentation, 50% (v/v) acetic acid or an alternative acid providing an equivalent carbon amount was applied as a consumption-based feed. The feed addition was controlled by the pH–stat function of the bioreactor system, where VFA uptake by *C. oleaginosus* was reflected by an increase in pH, triggering the automatic addition of the corresponding VFA feed to maintain the pH at the defined set point. The fed VFAs were supplied as free acids, while the initial VFA carbon sources in the medium were supplied as their corresponding sodium salts. The same pH-based feeding strategy and carbon-equivalent approach were applied for all tested VFAs, ensuring comparable substrate availability among the different fermentations. The initial optical density at 600 nm (OD_600_) was 0.5. Therefore, 150 mL of fermentation medium was inoculated with 1 mL of washed preculture. To monitor cell density, lipid accumulation, and potential contamination, fermentation broth from each reactor was examined every 24 h using an Axio Lab.A1 microscope equipped with an Axiocam 305 color (Carl Zeiss AG, Oberkochen, Germany). Samples were collected throughout the fermentation to determine OD_600_, dry cell weight, sugar concentration and fatty acid composition.

### Analytical methods

#### OD_600_ measurement

Cell growth was determined by measuring OD_600_ with a UV-3100PC spectrophotometer (VWR, Radnor, PA, USA). Samples from each bioreactor were analyzed in technical triplicate and diluted with phosphate-buffered saline to obtain OD_600_ values within the linear range of 0.1–1.0.

#### Dry cell weight

For the determination of dry cell weight (DCW), 3 mL of fermentation broth was transferred into pre-weighed tubes, mixed with 3 mL ethanol ≥99% (v/v), and centrifuged at 4400 rcf and 20 °C for 12 min. The resulting cell pellets were resuspended in 6 mL of 50% (v/v) aqueous ethanol and centrifuged again at 4400 rcf and 20 °C for 12 min. After discarding the supernatant, the samples were frozen and subsequently lyophilized. All samples were analyzed in technical duplicates.

#### Sugar and volatile fatty acid analysis

Sugars and volatile fatty acids in the fermentation media were quantified by high-performance liquid chromatography (HPLC) using an Agilent 1260 Infinity II LC system (Agilent Technologies, Waldbronn, Germany) equipped with a diode-array detector (DAD) and a refractive index detector (RID). Separation was performed on a Rezex ROA-Organic H+ (8%) column (Phenomenex, Torrance, CA, USA) using 5 mM H₂SO₄ as the mobile phase under isocratic conditions at a flow rate of 0.5 mL/min for 40 min, with the column oven maintained at 70 °C. RID detection was conducted at 40 °C [31]. Prior to analysis, the samples were prepared as described in Schneider et al.: samples were centrifuged at 15,000 rcf for 10 min, and the supernatant was stored at –20 °C. After thawing, samples were transferred to 10 kDa spin columns and filtered by centrifugation at 13,000 rcf and 20 °C. Finally, 197 µL of the filtrate was mixed with 3 µL of 0.5 M EDTA and analyzed by HPLC [14].

#### Fatty acid composition

The fatty acid composition of lyophilized cell biomass was determined by gas chromatography with flame ionization detection (GC-FID) following fatty acid methyl ester (FAME) derivatization, as described by Schneider et al. [14]. As an internal standard, either glyceryl trinonadecanoate (C19:0 TAG; Sigma-Aldrich, St. Louis, MO, USA) or glyceryl tridodecanoate (C12:0 TAG; Sigma-Aldrich, St. Louis, MO, USA) was used. Glyceryl trinonadecanoate was used for fermentations with even-chain VFAs, whereas glyceryl tridodecanoate was used for fermentations with odd-chain VFAs to avoid potential interference with the quantification of odd-chain fatty acids. Retention time calibration and quantification were performed using commercial FAME standard mixtures (Marine Oil Standard, 20 components, C14:0 to C24:1; Restek GmbH, Bad Homburg, Germany and Supelco 37 Component FAME Mix, Sigma-Aldrich, St. Louis, MO, USA). The lipid titer was calculated based on the quantified fatty acid composition obtained by GC-FID.

#### NMR

For NMR analysis, samples previously used for FAME detection were utilized. The derivatized samples were dried under high vacuum (≈2 mbar, 24 h) to remove residual volatile solvents and subsequently dissolved in CDCl₃ to a concentration of 5 mg/mL. NMR spectra were recorded at 298 K on Bruker AV-III-400 spectrometers. Proton and carbon chemical shifts (δ) are reported in ppm relative to tetramethylsilane (TMS) and were referenced internally to the residual solvent signals of CDCl₃. The following abbreviations are used for signal multiplicities: br = broad, s = singlet, d = doublet, t = triplet, p = pentet, m = multiplet.

#### Data analysis

All experiments were performed in biological triplicates unless otherwise stated. Data are presented as mean values ± standard deviation. Biomass concentration, lipid content, lipid titer, and fatty acid composition were calculated based on the analytical methods described above. Carbon-equivalent calculations were performed to ensure comparable carbon input across all tested substrates. Initial data processing and calculations were performed using Microsoft Excel (Microsoft Corporation, Redmond, WA, USA). Additional data sorting and processing, as well as the calculation of FAME compositions, were performed using Python (Python Software Foundation, Wilmington, DE, USA) where appropriate. Figures were prepared using OriginPro 2025 (OriginLab Corporation, Northampton, MA, USA).

## Results and discussion

### Experimental overview

To investigate the impact of volatile fatty acids (VFAs) on cell growth, lipid production, and fatty acid composition in the oleaginous yeast *C. oleaginosus*, systematic fed-batch fermentations were performed using acetic, propionic, butyric, valeric, and caproic acid, as well as the branched-chain acids 2-methylpropionic, 2-methylbutyric, and 3-methylbutyric acid. All experiments were performed on a carbon-equivalent basis, with acid concentrations in both the medium and the feed adjusted to ensure identical total carbon input. In addition, 3% (w/v) glucose was included in each fermentation to support stable initial yeast growth. To assess the influence of carbon chain length, an initial experimental series was conducted using straight-chain acids ranging from C2 to C6 (acetic to caproic acids). Dry cell weight, lipid titer, and fatty acid composition were determined from samples collected after 24, 48, 72, and 94 h of cultivation. In a second experimental series, the branched-chain acids (2-methylpropionic, 2-methylbutyric, and 3-methylbutyric acid) were evaluated in the same experimental setup. All experimental conditions were performed in biological triplicate.

### Chain length affects biomass formation and lipid allocation

**Fig. 1:**
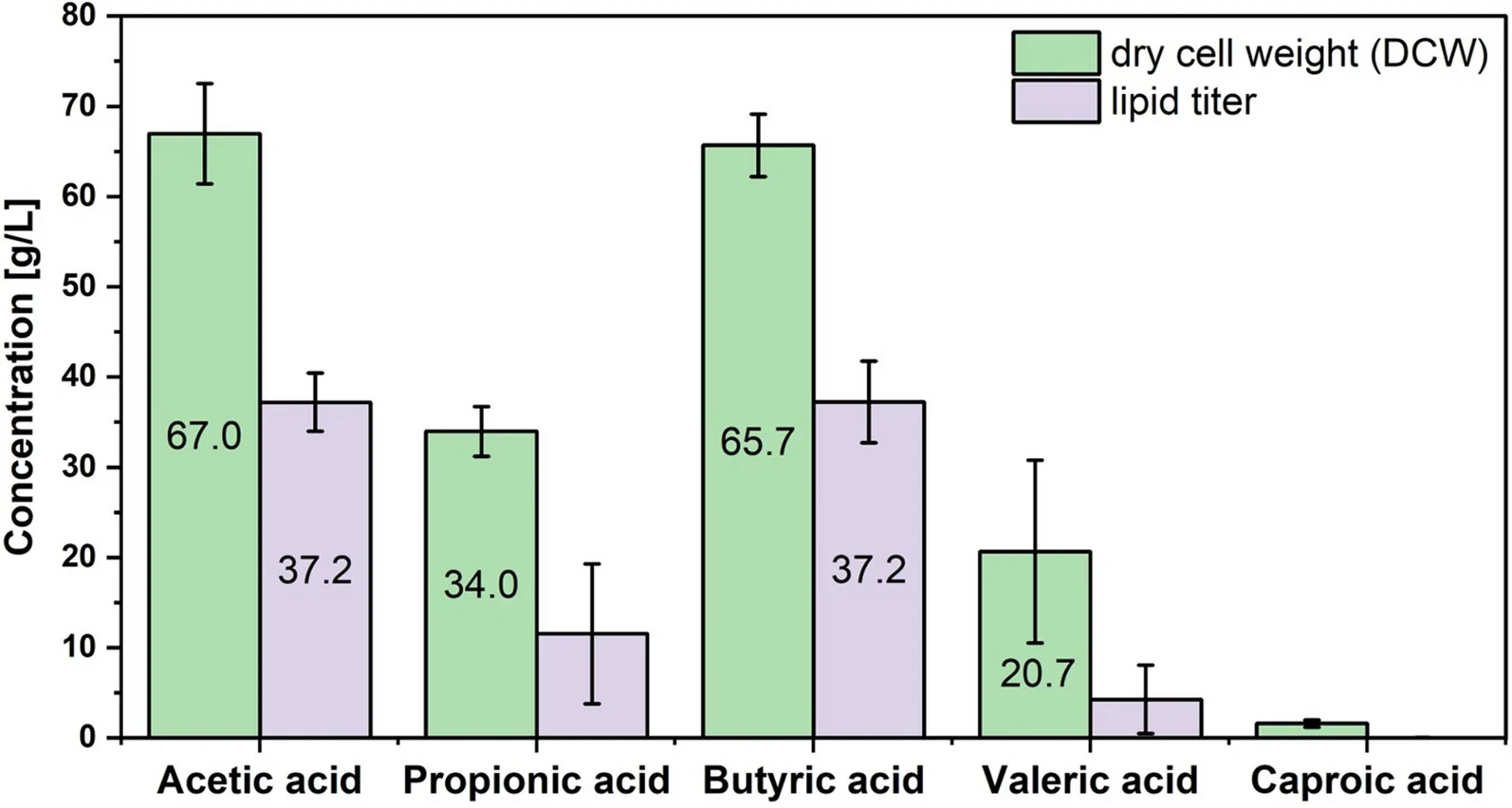
Dry cell weights (DCWs) and lipid titers of fed-batch fermentations using different volatile fatty acids as feed after 94 h. The tested acids included acetic (C2), propionic (C3), butyric (C4), valeric (C5), and caproic (C6) acids. Values are presented as mean ± standard deviation of biological triplicates (*n* = 3 for each acid)

To assess the influence of VFA chain length on process performance, C2–C6 substrates were compared for biomass formation and lipid accumulation (Fig. 1). After 94 h, the measured DCW ranged from 1.6 to 67.0 g/L (0.02–0.71 g/L/h) while lipid titer varied from 0.0 to 37.2 g/L (0.00–0.40 g/L/h). All DCW values over time are shown in the Supplementary Information Figure S1. These values indicate that the chain length of the fed VFA affects biomass formation and lipid allocation. The highest DCW and lipid titers were achieved with acetic acid and butyric acid feeding, reaching 67.0 g/L (0.71 g/L/h) and 65.7 g/L (0.70 g/L/h) DCW, respectively, with identical lipid titers of 37.2 g/L (0.40 g/L/h). These biomass and lipid production values obtained in fed-batch cultivations of *C. oleaginous* using acetic acid as a feed are consistent with those reported in the literature for acetic acid-based fermentations [13, 14, 32].

While acetic acid fermentation is well characterized in the literature, there are currently no reported values for dry cell weight (DCW) or lipid titer for the other acids tested here, as they have not previously been used individually as the main carbon sources in fermentation studies using the yeast *C. oleaginosus*. Most prior studies have focused on mixtures of volatile fatty acids (VFAs) and did not employ fed-batch fermentation strategies as used in this work, making direct comparisons difficult [28, 33–35]. This highlights the relevance of studying systematically VFAs to better understand their specific impact on growth and lipid accumulation in *C. oleaginosus*. In these experiments, propionic acid-based fermentation yielded a DCW of 33 g/L (0.35 g/L/h) and a lipid titer of 11.5 g/L (0.12 g/L/h), while valeric acid-based fermentation yielded a DCW of 20.7 g/L (0.22 g/L/h) and a lipid titer of 4.3 g/L (0.05 g/L/h). Fed-batch fermentation with caproic acid as a carbon source had a DCW of 1.6 g/L (0.02 g/L/h) and a lipid titer of 0.0 g/L (0.0 g/L/h) after 94 h, indicating nearly no growth and no lipid production.

Even-numbered straight-chain acids supported higher dry cell weights (DCW) and lipid titers than odd-chain acids, at least up to a chain length of C5. For caproic acids with a chain length of six carbon atoms, biomass formation was severely impaired, and no lipid production was observed, indicating a clear limitation in substrate utilization. Among the odd-chain acids, C3 (propionic acid) promoted higher biomass and lipid accumulation than C5 (valeric acid), suggesting a preference for shorter-chain substrates within this group. In general, increasing carbon chain length led to a progressive decline in both growth and lipid production, with butyric acid being the only substrate to yield results comparable to those of acetic acid.

The observed decline in growth and lipid production with increasing chain length could be attributed to enhanced substrate toxicity and limitations in cellular uptake. As carbon chain length increases, volatile fatty acids become more hydrophobic, a property that has been reported to promote cellular stress and toxicity while simultaneously impairing efficient substrate uptake [36]. While all fermentations were performed under identical, pH-controlled conditions, a minor contribution of undissociated VFA toxicity, particularly for longer-chain VFAs, cannot be excluded. Undissociated VFAs can diffuse across the cell membrane and dissociate within the cytosol, causing intracellular acidification and an increased energetic demand for maintaining pH homeostasis [37]. Consequently, the observed growth inhibition may reflect both acid stress and substrate-specific metabolic characteristics. The combined effects of increased membrane-associated toxicity, intracellular acid stress, and reduced intracellular substrate availability likely contributed to the decreased biomass formation and lipid production observed for longer-chain acids. Consistent with this, no glucose consumption was observed in the presence of caproic acid, indicating a strong inhibitory effect on cellular metabolism and suggesting pronounced substrate toxicity (Supplementary Information Figure S2). Notably, a different trend has been reported for the yeast *Yarrowia lipolytica*, where biomass formation increased with increasing chain length at low VFA concentrations, with the highest yield observed for caproic acid and the lowest for acetic acid. However, longer-chain VFAs were also associated with extended lag phases and increased toxicity at higher concentrations [38].

In *C. oleaginosus*, biomass formation and lipid accumulation showed a clear dependence on the carbon source provided. Interestingly, the volatile fatty acids with chain lengths of C2 (acetic acid) and C4 (butyric acid) yielded higher amounts of lipids than lipid-free biomass, whereas all other tested acids produced more lipid-free biomass than lipids (Fig. 1). This suggests that acetic acid and butyric acid are particularly effective substrates for volatile fatty acid-based fed-batch fermentations for lipid production. Their superior performance may be linked to their efficient conversion to acetyl-CoA, a direct precursor of fatty acid synthesis. In contrast, longer-chain or odd-chain acids likely require additional activation steps or generate intermediates that are less efficiently channeled into lipid biosynthesis. Moreover, increasing chain length is associated with higher hydrophobicity, which can enhance substrate toxicity, induce cellular stress, and impair substrate uptake, further limiting growth and lipid production. [16].

Overall, an inverse relationship between carbon chain length and both biomass and lipid formation was evident, highlighting the superior performance of short-chain acids, particularly C2 and C4, as substrates for lipid production in *C. oleaginosus*. These findings emphasize that substrate chain length is a key determinant of growth efficiency and lipid yield in volatile acid-based fed-batch fermentations.

### VFA chain length and parity determine the fatty acid composition

**Fig. 2:**
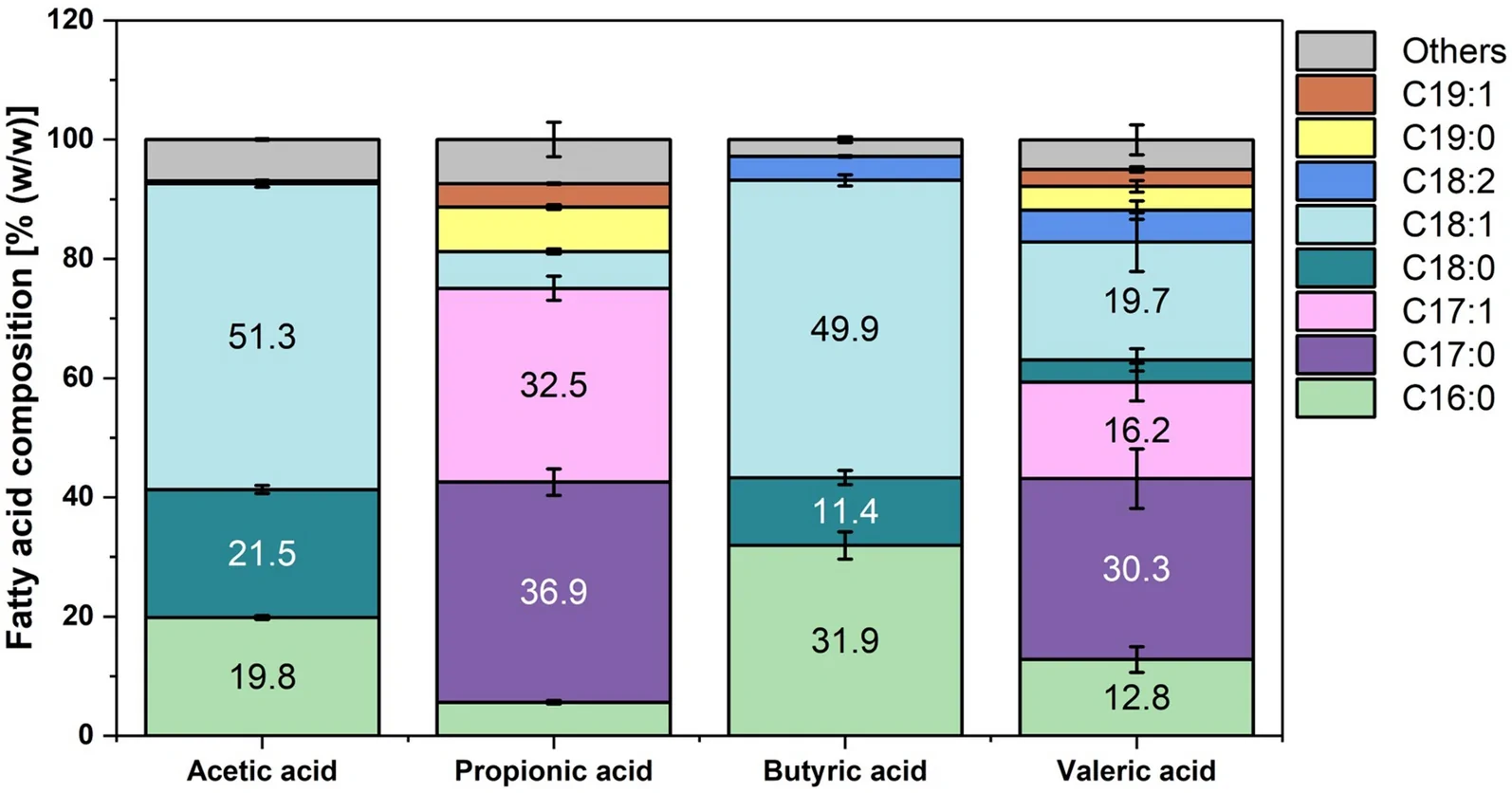
Fatty acid compositions (% (w/w)) after 94 h of fed-batch fermentation using acetic acid (C2), propionic acid (C3), butyric acid (C4) or valeric acid (C5) as feedstock. Values are presented as mean ± standard deviation of biological triplicates (*n* = 3 for each VFA)

While carbon chain length clearly influenced total lipid accumulation, it also affected the resulting fatty acid composition. The fatty acid profiles obtained after 94 h from fed-batch fermentations using different volatile fatty acids as feedstock are presented in Fig. 2. The fed-batch fermentation using caproic acid as the feed is not shown, as the lipid content remained below 2% (w/w), indicating negligible lipid formation.

A comparison of the fatty acid compositions obtained from fed-batch fermentations using acetic acid (C2), propionic acid (C3), butyric acid (C4), or valeric acid (C5) as feed clearly demonstrates distinct differences in both fatty acid profiles and relative abundances. Comparison of the fatty acid compositions from fermentations using the even-chain acids acetic acid and butyric acid revealed high levels of palmitic acid (C16:0), stearic acid (C18:0), and oleic acid (C18:1) in both cases (Fig. 2). Oleic acid (C18:1) was the most abundant, accounting for 51.3% in acetic acid-based fermentations and 49.9% in butyric acid-based fermentations. Notably, acetic acid fermentations yielded a higher proportion of stearic acid (C18:0, 21.5%) and a lower proportion of palmitic acid (C16:0, 19.8%). In contrast, butyric acid fermentations showed the opposite trend, with increased palmitic acid (C16:0, 31.9%) and decreased stearic acid (C18:0, 11.4%). Both substrates produced similar levels of oleic acid. The fatty acid compositions obtained from fermentations with acetic acid and butyric acid are likewise consistent with previously reported literature data for cultivations using these substrates [14, 33]. These results indicate that butyric acid fermentation favors the accumulation of palmitic acid at the expense of stearic acid compared to acetic acid, while the overall oleic acid content remains comparable.

By contrast, fermentations with odd-chain volatile acids exhibited fatty acid profiles that were strikingly different from those observed with even-chain substrates (Fig. 2). Both propionic acid (C3) and valeric acid (C5) fermentations were dominated by odd-chain fatty acids, particularly margaric acid (C17:0) and heptadecenoic acid (C17:1), along with smaller amounts of nonadecanoic acid (C19:0) and nonadecenoic acid (C19:1). Fermentation with propionic acid (C3) produced the highest levels of margaric acid (C17:0, 36.9%) and heptadecenoic acid (C17:1, 32.5%). Valeric acid (C5)-based fermentations also generated substantial amounts of these odd-chain fatty acids, with 30.3% margaric acid (C17:0) and 16.2% heptadecenoic acid (C17:1). While propionic acid (C3) resulted in only minor fractions of palmitic acid (C16:0) and oleic acid (C18:1), valeric acid (C5) fermentations contained more amounts of these even-chain fatty acids, including palmitic acid (C16:0, 12.8%) and oleic acid (C18:1, 19.7%), as well as small amounts of stearic acid (C18:0). The fatty acid composition observed in the fermentation using propionic acid is consistent with previously reported results from cultivations performed with propionic acid as the carbon source.[15, 33]. For example, Bonazini et al. reported a fatty acid profile containing approximately 34% margaric acid (C17:0) and 30% heptadecenoic acid (C17:1) after cultivation on 15 g/L propionic acid in shake flasks [15]. In the present study, even higher proportions of odd-chain fatty acids were achieved, likely due to the fed-batch fermentation strategy compared with shake flask cultivation. In contrast, no comparable literature on fatty acid profiles from fermentations using valeric acid as the primary carbon source has been identified to date.

A comparison of the proportions of even- and odd-chain fatty acids produced in fermentations using propionic acid (C3) or valeric acid (C5) as carbon sources revealed distinct product distributions. Propionic acid as a substrate resulted in 89.4% odd-chain fatty acids, whereas valeric acid led to a lower fraction of 55.9% odd-chain fatty acids (Supplementary Information Figure S3). Since 3% (w/v) glucose was used as a starter carbon source in all fermentations, the observed 11.6% fraction of even-chain fatty acids in the propionic acid fermentation can likely be attributed to glucose metabolism. It is well established that glucose metabolism leads to a fatty acid profile enriched in even-chain fatty acids, predominantly palmitic acid (C16:0), stearic acid (C18:0), and oleic acid (C18:1) [15]. This is further supported by the time-resolved fatty acid profiles (Supplementary Information Figure S4a and S4b), which show that these even-chain fatty acids are primarily formed within the first 24 h of fermentation, whereas odd-chain fatty acids are produced predominantly at later stages, coinciding with ongoing propionic acid feeding. Accordingly, a similar contribution of glucose-derived even-chain fatty acids can be assumed for the valeric acid fermentation. Although glucose contributes to the formation of even-chain fatty acids, its presence is essential in acid-fed fermentations, as it supports initial biomass formation and mitigates the inhibitory effects of volatile fatty acids on yeast growth [39]. However, these results indicate that, for the targeted production of odd-chain fatty acids, propionic acid is the more favorable substrate. It not only increases the fraction of odd-chain fatty acids but also supports higher lipid titers and improved cell growth compared to valeric acid.

**Fig. 3:**
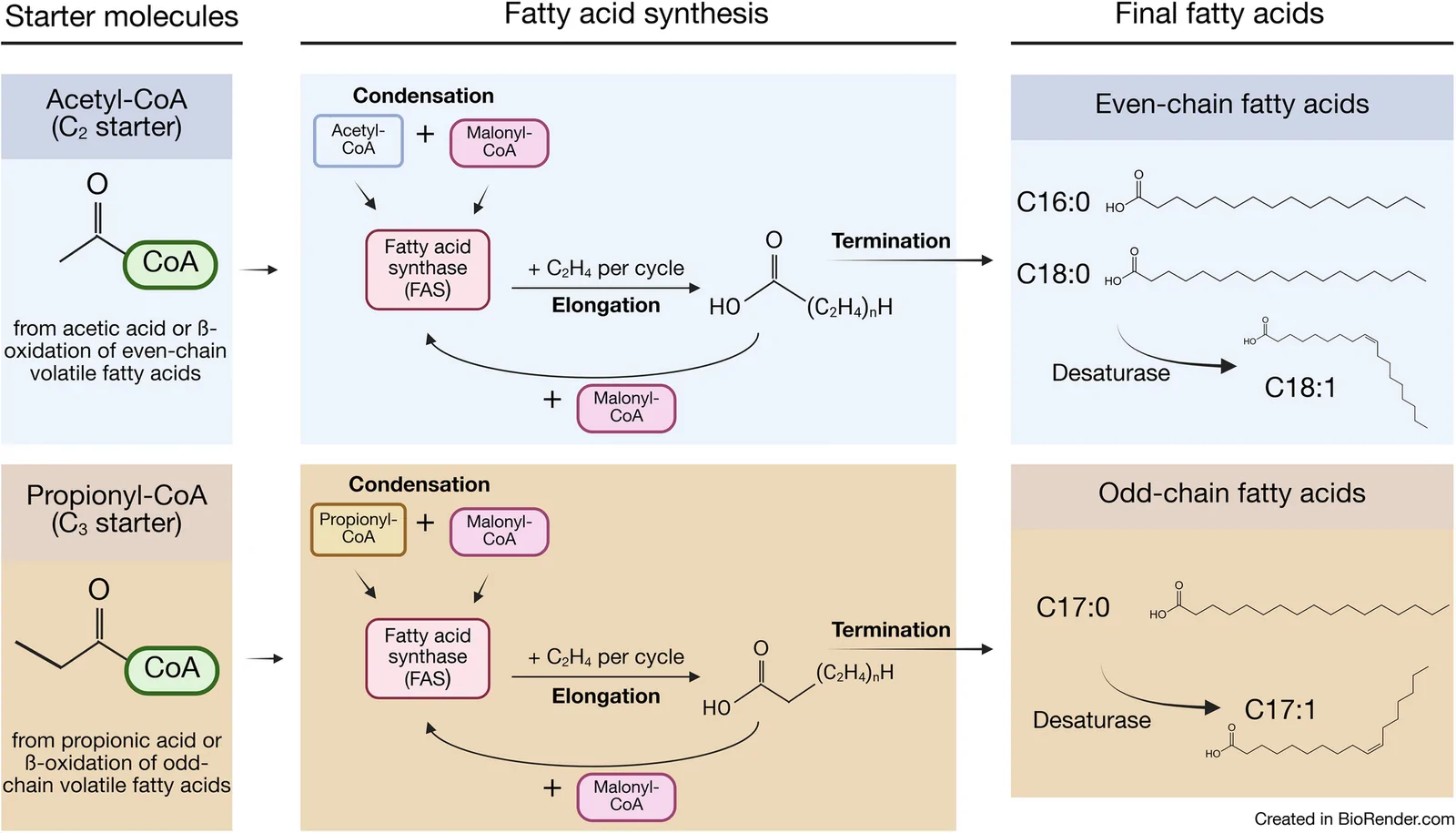
The starter molecules acetyl-CoA or propionyl-CoA determine the final fatty acid composition. Acetyl-CoA is derived either directly from acetic acid or from the β-oxidation of even-chain volatile fatty acids, whereas propionyl-CoA originates either from propionic acid or from the β-oxidation of odd-chain volatile fatty acids. These starter molecules undergo condensation with malonyl-CoA, catalyzed by the fatty acid synthase (FAS) complex. During each elongation cycle, the carbon backbone is extended by two carbon atoms through the incorporation of one malonyl-CoA molecule and simultaneous release of carbon dioxide. When acetyl-CoA serves as the starter molecule, the major products are typically palmitic acid (C16:0), stearic acid (C18:0), and, after desaturation, oleic acid (C18:1). In contrast, when propionyl-CoA is used as the starter molecule, the predominant products are heptadecanoic acid (C17:0) and its desaturated form heptadecenoic acid (C17:1), which is formed by the action of a desaturase enzyme

Odd-chain VFAs primarily led to the formation of odd-chain fatty acids, particularly margaric acid (C17:0) and heptadecenoic acid (C17:1). This can be explained by the nature of the starter molecules involved in fatty acid biosynthesis (Fig. 3). The short even-chain volatile fatty acid, acetic acid, gives rise to even-chain fatty acids because acetyl-CoA serves as the initiating substrate for fatty acid synthesis. Longer even-chain volatile fatty acids, such as butyric acid, are converted into acetyl-CoA via β-oxidation and can therefore also act as precursors for even-chain fatty acid formation. In contrast, odd-chain volatile fatty acids, such as propionic acid, are converted to propionyl-CoA, which serves as a precursor for odd-chain fatty acid synthesis. Similarly, longer odd-chain volatile fatty acids can be degraded through β-oxidation to yield propionyl-CoA as the starter molecule [15, 40, 41].

Both acetyl-CoA and propionyl-CoA are subsequently incorporated into the fatty acid synthase (FAS) complex, which catalyzes de novo fatty acid biosynthesis. In the initial condensation step, the respective starter molecule reacts with malonyl-CoA. During subsequent elongation cycles, the FAS complex extends the growing fatty acid chain by 2 carbon atoms per cycle by incorporating malonyl-CoA, accompanied by CO₂ release and NADPH consumption. Chain elongation continues until termination is initiated, resulting in the release of a fatty acid with a defined chain length. Consequently, the parity of the final fatty acid is determined by the starter molecule used for chain initiation. Acetyl-CoA-derived synthesis results in even-chain fatty acids, whereas propionyl-CoA-derived synthesis yields odd-chain fatty acids. Acetyl-CoA-based synthesis predominantly produces palmitic acid (C16:0), stearic acid (C18:0), and oleic acid (C18:1), with the unsaturated fatty acid being formed by subsequent desaturation through a desaturase enzyme. In contrast, propionyl-CoA primarily leads to fatty acids with a chain length of C17, predominantly occurring as either saturated margaric acid (C17:0) or monounsaturated heptadecenoic acid (C17:1), the latter likewise formed via desaturase activity (Fig. 3) [15, 40, 42].

At present, the use of valeric acid (C5) as a carbon source has not been described in the literature, and its impact on lipid biosynthesis in *C. oleaginosus* has not yet been systematically investigated. However, it can be assumed that both acetyl-CoA and propionyl-CoA contribute as precursors for lipid biosynthesis. This is supported by the observed composition of 55.9% odd-chain fatty acids and 44.1% even-chain fatty acids. Interestingly, the most abundant fatty acids in valeric acid fermentations combine those predominant in propionic acid fermentations (C17:0 and C17:1) with those predominant in acetic and butyric acid fermentations (C16:0, C18:0, and C18:1) (Fig. 2). It is likely that valeric acid undergoes partial β-oxidation, generating two- and three-carbon fragments that can then enter fatty acid biosynthesis as acetyl-CoA and propionyl-CoA. These mechanistic aspects require further investigation, potentially through targeted metabolomics studies, to elucidate the precise pathways involved.

To sum up, both the parity and the carbon chain length of VFAs play a key role in determining the fatty acid composition of lipids produced by *C. oleaginosus*. Even-chain VFAs, such as acetic acid and butyric acid, led to even-chain fatty acids, whereas the odd-chain VFA propionic acid resulted in mainly odd-chain fatty acids. Valeric acid (C5), an odd-chain VFA, produced a mixture of odd- and even-chain fatty acids, likely due to the combined contribution of acetyl-CoA and propionyl-CoA as starter molecules. These results indicate that by selecting specific VFAs as substrates, the fatty acid profile of the produced lipids can be tailored.

### Structural effects of branched-chain VFAs

**Fig. 4:**
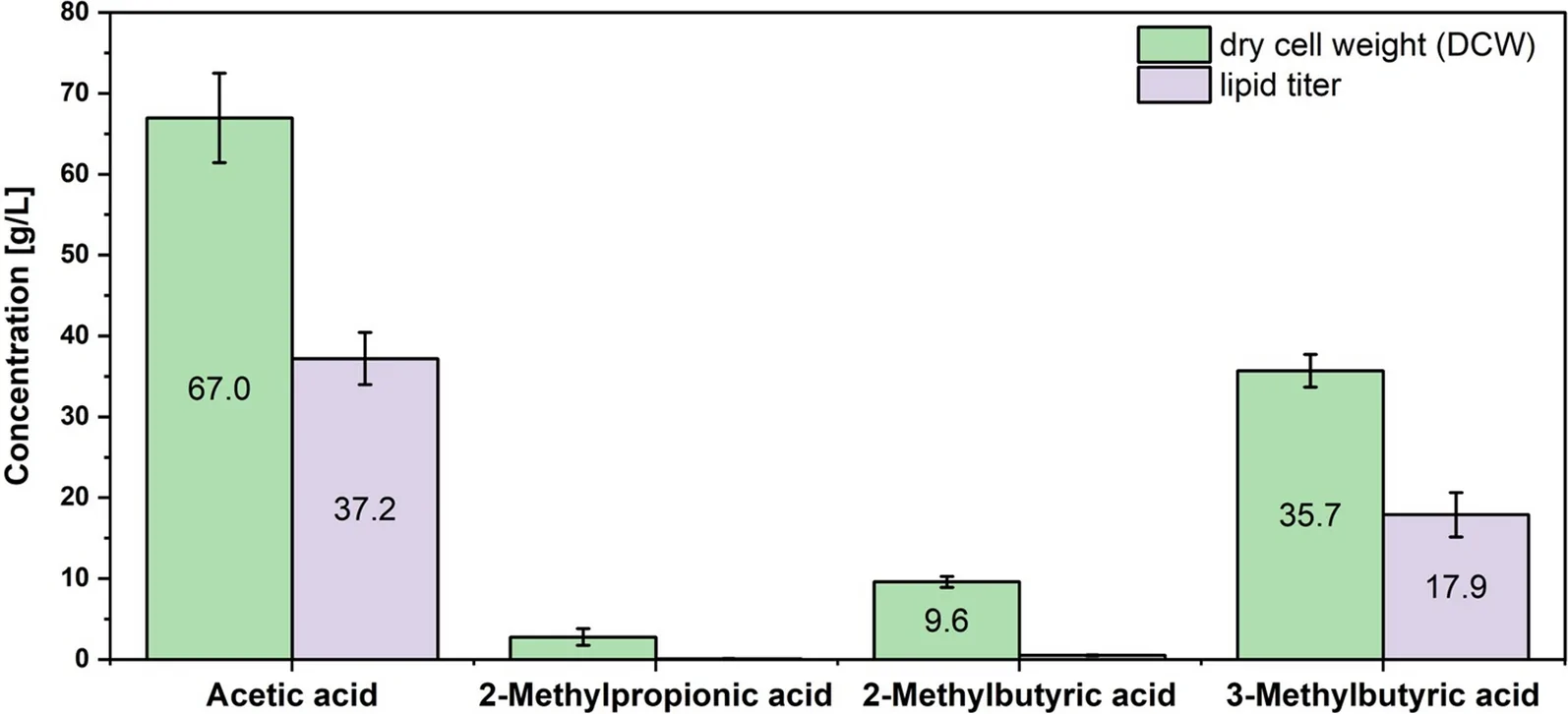
Dry cell weights (DCWs) and lipid titers of fed-batch fermentations using acetic acid or different branched volatile fatty acids as carbon feeds after 94 h. The tested branched volatile fatty acids were 2-methylpropionic acid, 2-methylbutyric acid, and 3-methylbutyric acid. Values are presented as mean ± standard deviation of biological triplicates (*n* = 3 for each acid)

In a second experimental series, the branched-chain acids 2-methylpropionic acid, 2-methylbutyric acid, and 3-methylbutyric acid were investigated in consumption-based fed-batch fermentations to assess the influence of a methyl substituent on lipid production in *C. oleaginosus*. Figure 4 presents the resulting dry cell weights (DCWs) and lipid titers obtained from these fermentations after 94 h. All DCW values over time are shown in the Supplementary Information Figure S5.

As shown in Fig. 4, methyl substitution markedly affected cell growth compared to the non-branched VFAs described above. Overall, methylated substrates resulted in reduced biomass formation. The highest dry cell weight (DCW) was achieved in fed-batch fermentations using 3-methylbutyric acid (isovaleric acid), reaching 35.7 g/L (0.38 g/L/h). In contrast, fermentations with methyl substitution at the C2 position (α-carbon) resulted in substantially lower biomass concentrations, with DCWs of 2.77 g/L (0.03 g/L/h) for 2-methylpropionic acid (isobutyric acid) and 9.59 g/L (0.10 g/L/h) for 2-methylbutyric acid. Furthermore, fed-batch fermentations with 2-methylpropionic acid and 2-methylbutyric acid resulted in almost no lipid accumulation, with lipid contents of only 2.8% and 5.2%, respectively. In contrast, fermentation with 3-methylbutyric acid led to a high lipid content of 50.1%, which is comparable to the lipid contents observed in acetic acid-based (55.6%) and butyric acid-based (56.7%) fermentations. The corresponding lipid titer for 3-methylbutyric acid reached 17.82 g/L (0.19 g/L/h). It should be noted that, compared to acetic acid and butyric acid, the tested branched-chain VFAs differ not only in carbon chain length but also in their structural configuration due to methyl substitution. These factors may affect substrate utilization and metabolic conversion, potentially contributing to the reduced biomass formation and lipid accumulation observed for the methylated substrates.

The HPLC data (Supplementary Information Figure S6) show that glucose uptake was almost completely inhibited during feeding with 2-methylpropionic acid. Since the fermentations were performed in fed-batch mode, only low concentrations of the respective VFAs were detected throughout the cultivation. Nevertheless, the HPLC analysis indicates a pronounced toxic effect of 2-methylpropionic acid, as the cells were unable to efficiently consume glucose, unlike the other branched VFAs tested.

Next to the toxic effects of 2-methylpropionic acid, it can be concluded that the position of the methyl group strongly influences both cell growth and lipid production. When the methyl group is located at the C2 position (α-carbon), both biomass formation and lipid accumulation are markedly reduced, whereas methyl substitution at the C3 position (β-carbon) allows substantial cell growth and lipid production. This effect may be explained by steric constraints during fatty acid biosynthesis. In the elongation cycle, the malonyl-ACP unit condenses with a starter unit, such as acetyl-CoA or propionyl-CoA, to initiate fatty acid formation. If the methyl group is positioned at the α-carbon (C2), it may sterically hinder the condensation reaction, thereby preventing efficient incorporation of the starter unit derived from the respective carbon source into the growing fatty acid chain. In contrast, when the methyl group is located at the β-carbon (C3), steric interference might be reduced, allowing the condensation reaction and subsequent chain elongation to proceed. These findings highlight that not only chain length and parity, but also molecular structure, play a crucial role in substrate selection and lipid biosynthesis.

**Fig. 5:**
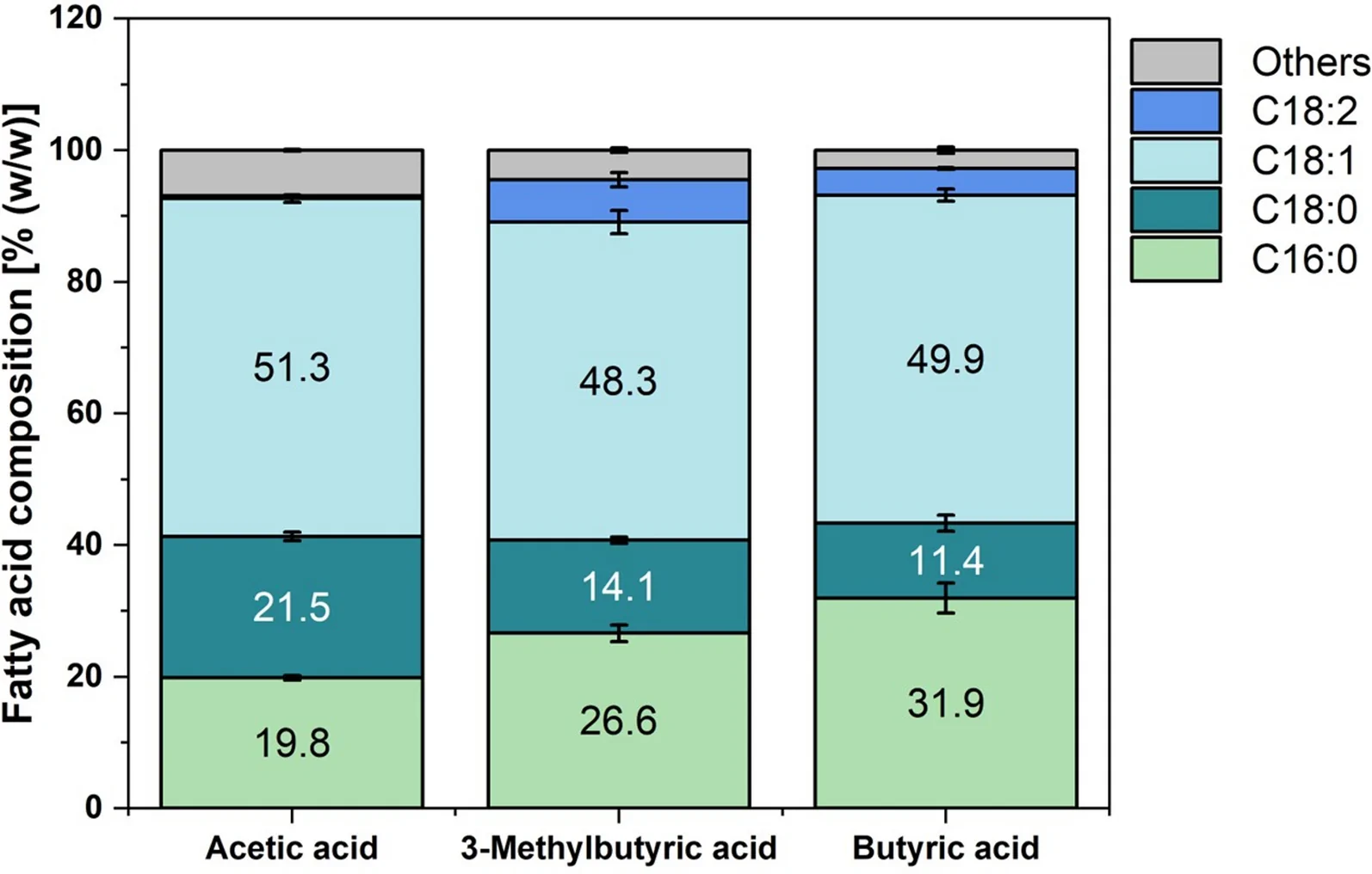
Fatty acid compositions (% (w/w)) after 94 h of fed-batch fermentation using acetic acid, 3-methylbutyric acid (isovaleric acid) and butyric acid as feedstock. Values are presented as mean ± standard deviation of biological triplicates (*n* = 3 for each acid)

A comparison of the fatty acid composition after 94 h obtained from 3-methylbutyric acid with that from acetic acid- and butyric acid-based fermentations reveals that the predominant components are similar (Fig. 5). The major fatty acids detected were palmitic acid (C16:0), stearic acid (C18:0), and oleic acid (C18:1), accompanied by smaller amounts of linoleic acid (C18:2) and other minor constituents. The fatty acid profile derived from 3-methylbutyric acid more closely resembles that observed in butyric acid-based fermentations, which is plausible given the identical backbone carbon chain length of both substrates. In 3-methylbutyric acid (isovaleric acid) fermentations, the relative proportions were 26.6% palmitic acid (C16:0), 14.1% stearic acid (C18:0), and 48.3% oleic acid (C18:1), compared to 31.9%, 11.4%, and 49.9%, respectively, in butyric acid-based fermentations. The fatty acid compositions of the fed-batch fermentations with 2-methylpropionic acid and 2-methylbutyric acid are not shown, as lipid production was negligible under these conditions (Fig. 4).

However, it remains unclear whether the methyl substituent is retained after fatty acid elongation. To clarify this, a more detailed analytical approach with higher chromatographic resolution is required to detect potential branched-chain fatty acids. If confirmed, the presence of methylated fatty acids could open up promising industrial applications, as branched-chain lipids often exhibit altered physicochemical properties, such as improved cold-flow behavior or oxidative stability, which may be advantageous for specialized bio-based products [43–45].

To gain deeper insight into the structural characteristics of the produced lipids, NMR analysis was performed using samples obtained from fed-batch fermentations with 3-methylbutyric acid after 94 h. Following lyophilization and derivatization, both ^1^H NMR (400 MHz, 300 K, CDCl3) and ^13^C NMR (400 MHz, 300 K, CDCl_3_) with 135-DEPT integration spectra were recorded (Supplementary Information Figure S6 and S7). The comprehensive NMR characterization suggests the formation of methyl (Z)-octadec-9-enoate (methyl oleate) as the major product. These data agree with literature values for methyl (Z)-octadec-9-enoate [46]. The cis-Δ9 configuration is assigned based on the characteristic olefinic chemical shift region (*δ* 5.3–5.4 ppm) and paired ^13^C olefinic resonances (130.0/129.8 ppm). The terminal methyl triplet (*δ* 0.88 ppm), the absence of iso-branched methine or septet signals, and the consistent chain integration confirm that the substrate-derived methyl group was not incorporated into the fatty acid backbone. The detected methyl group at the carbonyl position originates from the derivatization step and does not reflect structural modification of the fatty acid itself. These results suggest that methyl substitution in the substrate does not lead to significant incorporation of branched-chain precursors into the fatty acid fraction under the applied conditions, as no substantial changes in the overall fatty acid profile were observed compared to the corresponding linear substrate.In summary, the position of the methyl group is critical for both, cell growth and lipid production. In particular, 3-methylbutyric acid proved to be a promising substrate, enabling substantial biomass formation and lipid accumulation while yielding a fatty acid profile comparable to that obtained from the corresponding linear substrate.

### Industrial relevance of tailored fatty acid production

The fatty acid profiles obtained from even-chain volatile fatty acids consisted predominantly of palmitic acid (C16:0), stearic acid (C18:0), and oleic acid (C18:1), which are all commonly found in conventional vegetable oils such as palm, olive, or rapeseed oil. [9, 14]. Feeding with butyric acid resulted in a higher proportion of palmitic acid (C16:0) with 31.9% (19.8% for acetic acid feed) and a lower proportion of stearic acid (C18:0) with 11.4% (21.5% for acetic acid feed). The oleic acid (C18:1) content remained largely unchanged (49.9% butyric acid feed; 51.3% acetic acid feed) (Fig. 2). With this composition, it resembles palm oil even better, which has a composition of around 44% palmitic acid (C16:0), 4.5% stearic acid (C18:0), and 39.2% oleic acid (C18:1) [47]. Consequently, the microbial oils produced from these substrates could serve as potential alternatives to plant-derived oils and may be applied in the production of biofuels, lubricants, surfactants, and other oleochemicals [44, 48–51]. After overcoming regulatory barriers, these oils could also serve as sustainable lipid ingredients in the food sector [52].

In contrast, the odd-chain VFAs, propionic acid and valeric acid, promoted the formation of odd-chain fatty acids, particularly margaric acid (C17:0) and heptadecenoic acid (C17:1). Margaric acid (C17:0) naturally occurs mainly in ruminant fat and milk fat, typically at concentrations around 1%, and is only found in trace amounts in most vegetable oils [53, 54]. Similarly, heptadecenoic acid (C17:1) is mainly associated with ruminant-derived fats, milk fat, meat fat, and certain microbial sources, making both fatty acids comparatively rare in nature [40, 54, 55]. Thus, microbial production of odd-chain fatty acids offers not only higher product yields but also a more sustainable, environmentally friendly alternative to conventional sources. Compared to common even-chain fatty acids such as C18:0 and C18:1, odd-chain fatty acids exhibit altered physicochemical properties, including modified melting behavior and lipid packing characteristics, due to their asymmetric chain structure [56]. Moreover, odd-chain fatty acids are attracting increasing interest for specialized applications, as they have been associated with potential health benefits and represent promising precursors for high-value chemicals and tailored bio-based materials. In addition, they are explored as diagnostic biomarkers and as components in specialty lubricants and advanced oleochemical formulations. [40, 57, 58].

Although identical cultivation conditions were applied in the present study to enable a direct comparison of different VFAs, the results indicate that substrate-specific optimization may further expand the range of VFAs applicable for microbial lipid production. In particular, the conversion of caproic acid, 2-methylpropionic acid, and 2-methylbutyric acid may be enhanced through tailored process optimization, including controlled feeding strategies, co-feeding with readily metabolizable substrates such as acetic acid, adaptive laboratory evolution, or metabolic engineering targeting substrate uptake and acyl-CoA metabolism. These approaches may improve the utilization of longer-chain and branched-chain VFAs and further broaden the potential of VFA-based lipid production.

Despite the complexity of waste-derived VFA streams, the individual VFA fermentations performed in this study provide essential insights into the specific contribution of each compound to microbial lipid production. Real fermentation effluents typically contain mixtures of VFAs together with residual carbohydrates, alcohols, organic acids, nitrogen compounds, salts, and other potential inhibitors, which may influence substrate utilization and lipid accumulation [59]. The systematic evaluation of individual VFAs performed in this study enables the identification of substrate-specific effects related to carbon chain length, parity, and molecular structure. For example, mixtures enriched in even-chain VFAs may support the production of predominantly even-chain fatty acids, whereas the inclusion of odd-chain VFAs may enable the generation of odd-chain fatty acids and thereby expand the range of tailored lipid profiles. Furthermore, the different performance of branched-chain VFAs highlights that not only VFA composition but also molecular structure should be considered when designing mixed VFA feedstocks. Future studies should therefore investigate defined VFA mixtures based on waste-derived compositions as well as real waste-derived VFA streams to evaluate potential synergistic or inhibitory interactions under more complex conditions. These findings provide a basis for the rational design of VFA mixtures for tailored microbial lipid production. In particular, the use of propionic acid as a carbon source demonstrates the potential of VFA-based fed-batch fermentation for the targeted production of odd-chain fatty acids. Overall, these results highlight a sustainable strategy for converting low-value VFAs derived from waste streams into higher-value lipids with diverse industrial applications.

## Conclusion

To enable the rational use of specific VFAs for the production of microbial lipids with distinct and rarely occurring fatty acid profiles, this study established a systematic approach to investigate their effects on growth and lipid synthesis in *C. oleaginosus*. Since VFAs can be generated from diverse waste streams, their conversion into microbial lipids represents a sustainable alternative with broad industrial applicability, including oleochemicals, specialty lubricants, cosmetic ingredients, functional food components, and biofuels.

The results clearly demonstrate that substrate selection strongly affects both biomass formation and lipid accumulation. Furthermore, by carefully selecting substrates, it is possible to influence lipid composition and shift the lipid profile toward a desired direction. With increasing carbon chain length, growth and lipid production tended to decrease, with the highest biomass concentrations and lipid yields obtained in acetic acid- and butyric acid-based fermentations. In addition, substrate parity influenced the resulting fatty acid profile. Even-chain volatile fatty acids led to even-chain fatty acids (mainly C16:0, C18:0, and C18:1), whereas odd-chain volatile fatty acids promoted the formation of odd-chain fatty acids (mainly C17:0 and C17:1). This dependency can be attributed to the respective starter molecules, acetyl-CoA and propionyl-CoA, involved in fatty acid biosynthesis. Notably, C17:0 and C17:1 are of increasing interest due to their reported health relevance and their potential application as specialty oleochemicals with distinct physicochemical properties compared to conventional even-chain fatty acids. Owing to their low abundance in nature, these compounds represent high-value target products with significant industrial potential. For the targeted production of odd-chain fatty acids, propionic acid proved the most favorable substrate.

Experiments with branched volatile fatty acids further demonstrated that substrate structure is also crucial. When the methyl group was positioned too close to the carbonyl group, both growth and lipid production were strongly inhibited. In contrast, 3-methylbutyric acid enabled substantial biomass formation and high lipid accumulation, indicating its potential as a promising substrate. Importantly, the methyl group was not incorporated into the fatty acid backbone, resulting in a fatty acid composition comparable to that obtained with linear butyric acid.

Overall, these findings highlight the potential of VFAs as sustainable and tunable feedstocks for the production of tailored microbial lipids.

## Supplementary Information


Supplementary Material 1 (DOCX 5326)

## Data Availability

Data used for validation are included within the manuscript and supplementary information files. Additional raw datasets are available from the corresponding author upon request.

## Abbreviations

DAD: Diode array detector
DCW: Dry cell weight
DO: Dissolved oxygen concentration
FAME: Fatty acid methyl ester
GC-FID: Gas chromatography with flame ionization detection
HPLC: High-performance liquid chromatography
Mt: Metric Tons
NMR: Nuclear magnetic resonance
OD: Optical density
RID: Refractive index detector
SCO: Single-cell oil
TAG: Triacylglyceride
TMS: Tetramethylsilane
VFA: Volatile fatty acid
