# Supplementary material for: Volatile fatty acids as sustainable feedstocks for tailored microbial lipid production
Source: Biotechnol Biofuels Bioprod. 2026 Aug 28;19:66. doi: 10.1186/s13068-026-02814-x (PMC13525676; doi:10.1186/s13068-026-02814-x)
Supplement: Supplementary file 1 [file 13068_2026_2814_MOESM1_ESM.docx]

Supplementary Information

Research Paper

Volatile fatty acids as sustainable feedstocks for tailored microbial lipid production

Marieke Willing^1^, Max Schneider^1^, Mariia Kornilova^1^, Michel Weber^1^, Dania Awad^1^, Felix Melcher^1^, Michael Paper^1^, Marion Ringel^1*^, and Daniel Garbe^1*^

^1^ Werner Siemens-Chair of Synthetic Biotechnology, Technical University of Munich (TUM), TUM School of Natural Sciences, Lichtenbergstr. 4, 85748 Garching, Germany.

^*^Correspondence: Marion Ringel and Daniel Garbe, Werner Siemens-Chair of Synthetic Biotechnology, Technical University of Munich (TUM), TUM School of Natural Sciences, Lichtenbergstr. 4, 85748 Garching, Germany; Phone: +498928913250; Email: [marion.ringel@tum.de](mailto:marion.ringel@tum.de) and [daniel.garbe@tum.de](mailto:daniel.garbe@tum.de)

# Supporting Information

**Figure S1**. Dry cell weights (DCWs) of fed-batch fermentations using different volatile organic acids as feed over time. The tested acids comprised acetic, propionic, butyric, valeric, and caproic acids. Values are presented as mean ± standard deviation of biological triplicates (n = 3 for each acid).

**Figure S2**. Overview of the HPLC data from fed-batch fermentations using acetic acid (1), propionic acid (2), butyric acid (2), valeric acid (4), or caproic acid (5) as feed substrates. Shown are the concentration profiles of the respective acid and glucose over the course of fermentation. Values represent mean ± standard deviation of biological triplicates (n = 3 for each acid).

**Figure S3.** Comparison of the distributions of even- and odd-chain fatty acids produced in fermentations using acetic acid, propionic acid, butyric acid or valeric acid as carbon sources. Values are presented as mean ± standard deviation of biological triplicates (n = 3 for each acid).

**Figure S4a.** Fatty acid compositions (Fatty acid /DCW [g/g)]) after 24 h, 48 h, 72 h, and 94 h of fed-batch fermentation using acetic acid, propionic acid, butyric acid, or valeric acid as feed. Values are presented as mean ± standard deviation of biological triplicates (n = 3 for each VFA).

**Figure S4b.** Fatty acid compositions (% (w/w)) after 24 h, 48 h, 72 h, and 94 h of fed-batch fermentation using acetic acid, propionic acid, butyric acid, or valeric acid as feed. Values are presented as mean ± standard deviation of biological triplicates (n = 3 for each VFA).

**Figure S5.** Dry cell weights (DCWs) of fed-batch fermentations using acetic acid, 2-methylpropionic acid, 2-methylbutyric acid or 3-methylbutyric acid as a feed. Values are presented as mean ± standard deviation of biological triplicates (n = 3 for each acid).

**Figure S6**. Overview of the HPLC data from fed-batch fermentations using acetic acid (1), 2-methylpropionic acid (2), 2-methylbutyric acid (3) or 3-methylbutyric acid (4) as feed substrates. Concentrations of the respective acids and glucose are shown over cultivation time. Values represent mean ± standard deviation of biological triplicates (n = 3 for each acid).

**Figure S7.** ^1^H NMR (400 MHz, 300 K, CDCl_3_, referenced to the residual solvent signal at δ 7.26 ppm) of samples obtained from fed-batch fermentations with 3-methylbutyric acid after 94 h. The ^1^H NMR spectrum exhibits characteristic signals for a C18:1 fatty acid methyl ester: δ 5.43 – 5.27 (m, 2H, -CH=CH-, H-A Δ9-cis), 3.67 (s, 3H, -COOCH_3_, H-B), 2.30 (t, J = 7.6 Hz, 2H, -CH_2_-COOCH_3_, H-C), 2.09 – 1.96 (m, 2H, allylic, H-D), 1.63–1.60 (m, 4H, allylic H-D + CH_2_ H-E), 1.31 – 1.24 (br m, 20H, -(CH_2_)_10_- chain, H-F), and 0.88 ppm (t, J ≈ 7.0 Hz, 3H, CH_3_-CH_2_-, H-G).

**Figure S8**. ^13^C NMR (400 MHz, 300 K, CDCl_3_, referenced to the residual solvent signal at δ 77.16 ppm) with 135-DEPT integration of samples obtained from fed-batch fermentations with 3-methylbutyric acid after 94 h. The ¹³C NMR shows 19 distinct signals matching authentic methyl oleate: δ 174.3 (C=O), 130.0/129.8 (=CH), 51.4 (OCH_3_), 34.2 (α-CH_2_), 31.9–22.7 ((CH_2_)_n_), 14.1 (CH_3_). The DEPT-135 analysis confirms expected multiplicities with positive signals for CH/CH_3_ (130.0/129.8 ppm =CH, 51.4 ppm OCH_3_, 14.1 ppm CH_3_) and negative signals for CH_2_ groups (34.1 ppm α-CH_2_, 25–32 ppm aliphatic chain).
